# Improved Quantum Artificial Fish Algorithm Application to Distributed Network Considering Distributed Generation

**DOI:** 10.1155/2015/851863

**Published:** 2015-09-01

**Authors:** Tingsong Du, Yang Hu, Xianting Ke

**Affiliations:** ^1^Department of Mathematics, Science College, China Three Gorges University, Yichang 443002, China; ^2^Hubei Province Key Laboratory of System Science in Metallurgical Process, Wuhan University of Science and Technology, Wuhan 430081, China

## Abstract

An improved quantum artificial fish swarm algorithm (IQAFSA) for solving distributed network programming considering distributed generation is proposed in this work. The IQAFSA based on quantum computing which has exponential acceleration for heuristic algorithm uses quantum bits to code artificial fish and quantum revolving gate, preying behavior, and following behavior and variation of quantum artificial fish to update the artificial fish for searching for optimal value. Then, we apply the proposed new algorithm, the quantum artificial fish swarm algorithm (QAFSA), the basic artificial fish swarm algorithm (BAFSA), and the global edition artificial fish swarm algorithm (GAFSA) to the simulation experiments for some typical test functions, respectively. The simulation results demonstrate that the proposed algorithm can escape from the local extremum effectively and has higher convergence speed and better accuracy. Finally, applying IQAFSA to distributed network problems and the simulation results for 33-bus radial distribution network system show that IQAFSA can get the minimum power loss after comparing with BAFSA, GAFSA, and QAFSA.

## 1. Introduction

Distributed generation (DG) is small-scale and radial generating facilities, which are placed in the vicinity of the load, delivering electricity to consumers independently. Compared with the traditional and centralized power, DG has many advantages [1, 2] such as being near to the center of the users and having high energy efficiency and lower cost of construct. Studies show that DG grid connection has significant impact on the distributed network, including power flow, voltage profile, system losses, and reliability, the extend of which has something to do with the location of DG intimately [3–5]. On the one hand, the suitable installation position and capacity can improve the voltage quality effectively and reduce the active loss; on the other hand, unsuitable configuration turns out to be just the opposite wish and threat to the safe and stable operation of the power network. There are many approaches for deciding the optimum sizing and sitting of DG units in distribution systems. Some of them rely on conventional optimization methods and others use artificial intelligence-based optimization methods [6]. The new methods of distribution network programming, Genetic Algorithm (GA) [7, 8], Ant Colony Algorithm (ACA) [9], Particle Swarm Algorithm (PSA) [10], and Chaos Algorithm (CA), have advantages and disadvantages, respectively. The robustness of the GA is strong in addition to its ability to be trapped in a local minimum. Initial particles generated by CA have property of ergodicity but slower convergence speed. Therefore, how to effectively combine the merits of different intelligent algorithms to improve the performance among search algorithm is a worth studying direction [11–13].

Basic artificial fish swarm algorithm (BAFSA) is a kind of intelligence optimization algorithm based on animal behavior by Li et al. in 2002 [14]. According to the characteristics of the fish swarm and its animal autonomous model, it simulates behavior of fish to achieve the purpose of the group global optimization by each individual in the local optimization. The main fish behaviors are the following: foraging, huddling, following, and being random. In almost all the cases, the BAFSA is easy to avoid falling into local optimum and obtain the global optimum. Although the algorithm with the merits of strong robustness and good convergence performance is not sensitive to initial value and parameter selection, it has the weakness with search efficiency such as the poor ability to keep balance of exploration and development, late blind search, slow arithmetic speed, and low accuracy of optimization results.

Quantum computing that is different from the traditional calculation model of classical physics has incomparable advantages such as quantum super parallelism and exponential storage capacity [15]. The combination of quantum computing and intelligent optimization algorithm injected new life into the intelligent optimization computing source, by using quantum computing in a new mode of representing and processing information, and so forth [16, 17]. Intelligent optimization algorithm can be designed from another angel to enrich the theory of intelligent computing and improve the traditional method of intelligent search performance as a whole.

In the present paper, the improved quantum artificial fish swarm algorithm (IQAFSA) is proposed in consideration of the slow speed and low accuracy of BAFSA and randomness and blindness of quantum computing [18, 19]. The proposed algorithm improves the coding way of the quits artificial fish and uses quantum revolving door, artificial fish following, artificial fish preying, and variation of update strategy to complete self-renewal, resulting in a new artificial fish. IQAFSA is then applied to solve high dimensional and complex nonconvex programming and distributed network programming considering distributed generation. By simulation experiment among BAFSA, the global edition artificial fish swarm algorithm (GAFSA) has been put forward in [20] and quantum artificial fish swarm algorithm (QAFSA). The simulation results indicate that IQAFSA has great convergence and superiority in function optimization; meanwhile, they illustrate the validity and feasibility of IQAFSA applied to optimal configuration of DG in distributed network system.

The rest of this paper is organized as follows. We devote Section 2 to a discussion of those aspects of basic idea of BAFSA. The detail on quantum computing is then discussed in Section 3. The update of the quantum bit needs to make use of the transformation of quantum gates. In Section 4, the QAFSA has been formulated. The IQAFSA given in Section 5 is encoded by quantum bits and updated by quantum revolving gate. Artificial fish perform preying behavior and following behavior. In Section 6, the effectiveness of the algorithm compared with those of BAFSA, GAFSA, and QAFSA for three multidimensional and box constraints programming systems is demonstrated except for one two-dimensional box constraint programming.  Section 7 deals with the distributed network modeling and the effect of DGs on system losses. Finally, Section 8 is devoted to drawing of the conclusions.

## 2. Description of BAFSA

Artificial fish complete update and obtain the optimal value mainly through the following four behaviors: being random, preying, swarming, and following in the process of iterative calculation.

### 2.1. AF-Random

Random behavior is to randomly select a new state *x*_next_ in its visual field and then move a step in the direction. It is actually a default behavior.

### 2.2. AF-Prey

Preying is a kind of the basic behavior of artificial fish, which move to the direction of food with high concentration. The AF-preying behavior is described as follows:

(1) $$\begin{array}{c} X_{inext}=\left\{\begin{array}{cc} x_{ik}+\text{Random(step)}\frac{x_{jk}-x_{ik}}{\left\|X_{j}-X_{i}\right\|}, & Y\left(X_{j}\right)\text{ is better than }Y\left(X_{i}\right)\\ x_{ik}+\text{Random(step)}, & else, \end{array}\right. \end{array}$$

where *x*_*jk*_ is *k*th element of state vector; *X*_*j*_ is *k*th elements of *X*_*i*_; *x*_*i*next_ is the next step state vector; Random(step) is a random number between 0 and step *k* = 1,2,…, *n*.

### 2.3. AF-Swarm

AF-swarm refers to the fact that every fish moves to the center of the adjacent partners in the process of swimming as fast as possible and avoids overcrowding. Suppose that *X*_*i*_ is the current state of artificial fish *i*. *n*_*f*_ is the number of partners and *X*_*c*_ is the central position in the current field (*d*_*ij*_ < Visual). If *Y*_*c*_/*n*_*f*_ > *δY*_*i*_, it shows that there are more food around the partner and its not too crowded. Then, the fish moves a step forward to the central position of this partner. The AF-swarming behavior is described as follows:

(2) $$\begin{array}{c} X_{i}^{i+1}=X_{i}^{t}+\frac{X_{c}-X_{i}^{t}}{\left\|X_{c}-X_{i}^{t}\right\|}\cdot \text{step}\cdot Rand\left(\cdot \right). \end{array}$$

Otherwise, the fish performs preying behavior.

### 2.4. AF-Follow

AF-follow illustrates that each artificial fish moves to the current optimal direction in the range of vision. Suppose that *X*_*i*_ is the current state of artificial fish *i*. *Y*_*j*_ is the greatest partner in the current field (*d*_*ij*_ < Visual). If *Y*_*j*_/*n*_*f*_ > *δY*_*i*_, it turns out to be that there are more food around *X*_*j*_ while being not too crowded. Then, the fish moves a step forward to the direction of *X*_*j*_. The AF-following behavior is described as follows:

(3) $$\begin{array}{c} X_{i}^{t+1}=X_{i}^{t}+\frac{X_{j}-X_{i}^{t}}{\left\|X_{j}-X_{i}^{t}\right\|}\cdot \text{step}\cdot Rand\left(\cdot \right). \end{array}$$

Otherwise, the fish performs preying behavior.

In the problem of function optimization. Suppose that in a *D*-dimensional search space goal, the number of artificial fish is *n*. The state of the individual artificial fish can be denoted by vector *X* = (*x*_1_, *x*_2_,…, *x*_*n*_) and *x*_*i*_  (*i* = 1,2,…, *n*) which is the optimization variable. The food concentration of the artificial fish in the current location can be expressed as *Y* = *f*(*X*), where *Y* denotes the objective function value. *d*_*ij*_ = ‖*X*_*i*_ − *X*_*j*_‖ is the distance between the fish *i* and the fish *j*. The idea of BAFSA is that it firstly initializes a swarm of artificial fish randomly; secondly the fish complete update by four basic behaviors mentioned above. Each of the artificial fish explores the current environment conditions (including the change of the objective function and partners). Then, select an appropriate behavior and move in the direction of optimal areas. Finally, artificial fish gather around several local optimal, especially in some better optimal areas, which are generally able to rally more artificial fish, namely, the optimal value of objective function.

## 3. Quantum Computing

Physical media acting as the information storage unit are a two-state quantum systems in quantum computing, to be referred to as quantum bit. A quantum bit can be in a state of quantum superposition represented by |*φ*〉 = *α* | 0〉+|1〉, where *α* and *β* denote quantum bit probability amplitude. The probability of |0〉 is represented by |*α*|^2^, and the probability of |1〉 is represented by |*β*|^2^; furthermore, |*α*|^2^ + |*β*|^2^ = 1. Each quantum state in system can be expressed as the superposition of the basic state; as |*α*|^2^ and |*β*|^2^ approach to be 1 or 0, the quantum bit will converge to a specific state.

The update of the quantum bit needs to make use of quantum gates which include the xor gate, the controlled xor gate, Hadamard transform gate, and the revolving gate. The quantum revolving gate [21, 22] was adopted in this paper to change its phase by interference and the basic state probability amplitude. The revolving gate is described as follows:

(4) $$\begin{array}{c} U\left(\theta \right)=\left[\begin{array}{cc} \cos\left(\theta \right) & -\sin\left(\theta \right)\\ \sin\left(\theta \right) & \cos\left(\theta \right) \end{array}\right], \end{array}$$

where *θ* denotes the rotation angle of the quantum gate; the update process is as follows:

(5) $$\begin{array}{c} \left[\begin{array}{c} \alpha^{\prime }\\ \beta^{\prime } \end{array}\right]=U\left(\theta \right)\left[\begin{array}{c} \alpha \\ \beta \end{array}\right]=\left[\begin{array}{cc} \cos\left(\theta \right) & -\sin\left(\theta \right)\\ \sin\left(\theta \right) & \cos\left(\theta \right) \end{array}\right]\left[\begin{array}{c} \alpha \\ \beta \end{array}\right]. \end{array}$$

The rotation angle of quantum gate is adjusted dynamically with the evolutionary process, whilst rotation angle is set larger at the beginning of the algorithm. With the increase of evolution generation, the rotation angle is gradually reduced by formula *θ* = *k* · *s*(*α*_*i*_, *β*_*i*_), where *k* is a coefficient influencing the speed and *s*(*α*_*i*_, *β*_*i*_) is the direction of rotation angle. Emphasis here is on the fact that if *k* is very large, the search step length will be very big in each iteration in the process of calculation. It is easy to fall into local optimal value. On the other hand, if the convergence speed is slow, the computation time will be too long. In this paper, *k* = 10 · exp(−*t*/*T*), where *t* denotes the current evolution generation, and *T* is the biggest evolution generation. *s*(*α*_*i*_, *β*_*i*_) is to make the algorithm get the optimal solution, the principle of which is to use the current solution to approach to the best solution best_*i*_, determining the direction of rotation of the quantum revolving gate. An adjusting strategy which is general and has nothing to do with the problem [23] is adopted in this paper. These strategies are described in Table 1.

## 4. Description of QAFSA

The artificial fish is encoded with quantum bits in QAFSA. It mainly uses update strategy of quantum revolving gate for self-renewal to get new artificial fish. Compared with BAFSA, the diversity of its fish becomes richer. Despite the small scale of the fish, it does not affect the convergence of the algorithm. At the same time, the algorithm has faster convergence speed as well as higher accuracy.

Suppose that there is a quantum of artificial fish *Q*(*t*) = {*q*_1_^*t*^, *q*_2_^*t*^,…, *q*_*n*_^*t*^}, where *n* denotes the population size, *t* is evolution generation, and *q*_*j*_^*t*^ represents a quantum artificial fish, which is encoded by quantum bit as follows:

(6) $$\begin{array}{c} q_{j}^{t}=\left[\begin{array}{cccc} \alpha_{1}^{t} & \alpha_{2}^{t} & \cdots & \alpha_{m}^{t}\\ \beta_{1}^{t} & \beta_{2}^{t} & \cdots & \beta_{m}^{t} \end{array}\right],\;j=1,2,\ldots ,n, \end{array}$$

where *m* denotes the quantum number. An artificial fish encoding with multiple quantum bits can be represented as [19]
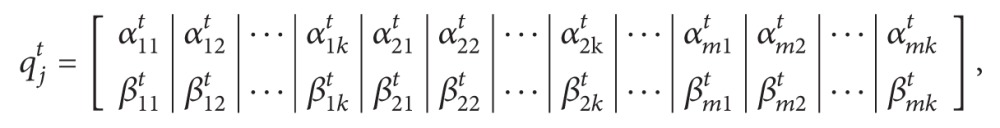
(7)
where *m* is variable dimension of artificial fish optimal control,  *k* is quantum bit number of optimal control variables, *α*_*xy*_ and *β*_*xy*_  (1 ≤ *x* ≤ *m*, 1 ≤ *y* ≤ *k*) are complex constants, and |*α*_*xy*_|^2^ + |*β*_*xy*_|^2^ = 1.

The quantum bits coding (*α*, *β*) of each individual is initialized to $\left(1/\sqrt{2},1/\sqrt{2}\right)$. A measurement is carried out for the initialization of the artificial fish in order to obtain a set of definite solution *P*(*t*) = *p*_1_^*t*^, *p*_2_^*t*^,…, *p*_*n*_^*t*^, where *p*_*j*_^*t*^ is *j*th solution in *t*th generation population, which is performed by binary string of length *m*, each of which is 0 or 1 and gotten by the probability of quantum bits. The measurement process is randomly generating a number between 0 and 1. If this number is greater than |*α*_*i*_^*t*^|^2^, the result value is 1, otherwise 0. In each iteration, firstly, measure the population to get a set of determinate solutions *P*(*t*), and then calculate the fitness value of each solution. Using quantum revolving gate to adjust individual in the population to obtain the updated population, record the current optimal solution, and compare with the current target value. If it is better than the current target, let the new optimal solution be a target for the next iteration. Otherwise, keep the current target value the same.

## 5. Description of IQAFSA

Despite the fact that QAFSA can reach high precision and convergence speed, it may get some inferior solutions while producing better one. The fish diversity is not very rich and the convergence speed can be further improved. Therefore, the IQAFSA given in this paper is encoded by quantum bits and updated by quantum revolving gate. Make it perform preying behavior and following behavior. And swap quantum probability amplitude of artificial fish having poor results can realize the variation, and then the optimal solution is obtained [24].

At the early stage of the quantum artificial fish optimization, each time the artificial fish has achieved an update, it will turn into the implementation of the tailgating behavior of AFSA, which can improve the convergence speed of artificial fish. At the late stage of the artificial fish quantum optimization, each time the artificial fish has realized an update, it will turn into the implementation of the preying behavior of AFSA, which can improve the accuracy of optimization. Having been updated by quantum revolving gate, preying behavior, and following behavior, for those artificial fish having poor optimization results, it can enrich the diversity of the artificial fish by swapping the quantum bit probability amplitude value, namely, (*α*, *β*), and reversing the individual evolutionary direction as a whole, which can prevent the individual evolution falling into a local optimum. Finally, the variation of artificial fish is brought about. Here a point that should be stressed is that the* Pauli*-*X* gate is used below to realize mutation operation:

(8) $$\begin{array}{c} \left[\begin{array}{cc} 0 & 1\\ 1 & 0 \end{array}\right]\left[\begin{array}{c} \alpha \\ \beta \end{array}\right]=\left[\begin{array}{c} \beta \\ \alpha \end{array}\right]. \end{array}$$

Because the quality of preying and following behavior has close relation with vision and step of artificial fish. The results in the literature [22] show that the larger range of vision is, the stronger global search ability of artificial fish is and faster convergence speed is. On the contrary, the smaller range of vision is, the stronger local search ability of artificial fish is. Whilst the larger size of step is, the faster convergence speed is; however, it will turn out to be an oscillation phenomenon. On the other hand, the smaller size of step is, the faster convergence speed is, but there will be the high precision of the solution. Consequently, the dynamic adjustments of artificial fish vision and step size are as follows:

(9) $$\begin{array}{c} \text{visual}=\text{visual}\cdot a+\text{visua}{\text{l}}_{\min},\\ \text{step}=\text{step}\cdot a+\text{ste}{\text{p}}_{\min},\\ a=\exp\left[-30\cdot {\left(\frac{t}{T}\right)}^{s}\right]. \end{array}$$

IQAFSA steps are as follows.

**Step 1 .**

Initialize the quantum of artificial fish *Q*(*t*_0_) = *q*_1_^*t*_0_^, *q*_2_^*t*_0_^,…, *q*_*n*_^*t*_0_^ in the feasible region, and all optimal control variables (*α*_*i*_^*t*_0_^, *β*_*i*_^*t*_0_^) of the artificial fish populations are initialized to $\left(1/\sqrt{2},1/\sqrt{2}\right)$. Set up parameters such as the artificial fish vision, step length, crowded degree, maximum attempts, and evolution generation.

**Step 2 .**

Calculate the fitness of each artificial fish in the population *Q*(*t*); the determinate solution *P*(*t*) can be calculated.

**Step 3 .**

Conduct fitness evaluation for the determinate solutions and put the optimal artificial fish and its fitness value in the bulletin board.

**Step 4 .**

On the basis of the above adjustment strategy, firstly use the quantum revolving gate *U*(*θ*) and the corresponding rotation angle adjustment policy to update quantum bit of artificial fish *Q*(*t* + 1), and perform preying behavior and following behavior for artificial fish through the fish evolution process. Finally, new artificial fish is gotten.

**Step 5 .**

Perform mutation for artificial fish, choose *q*  (*q* < *n*) artificial fish having minimum fitness, and update its quantum bit probability amplitude by using formula (6).

**Step 6 .**

If evolution generation achieves the maximum, then output the result on the bulletin board as the final optimal solution; otherwise, increase the iteration number by *g* = *g* + 1 and go back to Step 2.

## 6. Simulation Experiment and Analysis

In order to verify the feasibility and effectiveness of  IQAFSA, four typical function simulations are proposed compared with QAFSA, BAFSA, and GAFSA in the literature [20]:

 E.g. 1 [20]:
  (10) $$\begin{array}{c} min\;f_{1}\left(x\right)=\overset{n}{\underset{i=1}{\sum }}\left[x_{i}^{2}-10\cos\left(2\Pi x_{i}\right)+10\right]\\ s\text{.}t.\;-10<x_{i}<10 \end{array}$$
 E.g. 2 [20]:
  (11) $$\begin{array}{c} min\;f_{2}\left(x\right)=\frac{1}{4000}\overset{n}{\underset{i=1}{\sum }}x_{i}^{2}-\overset{n}{\underset{i=1}{\prod }}\cos\left(\frac{x_{i}}{\sqrt{i}}\right)+1\\ s\text{.}t.\;-100<x_{i}<100 \end{array}$$
 E.g. 3 [25]:
  (12) $$\begin{array}{c} min\;f_{3}\left(x\right)=-20\exp\left(-0.2\sqrt{\frac{1}{n}\overset{n}{\underset{i=1}{\sum }}x_{i}^{2}}\right)-\exp\left(\frac{1}{n}\overset{n}{\underset{i=1}{\sum }}\cos\left(2\Pi x_{i}\right)\right)+20+e\\ s.t.\;-32<x_{i}<32 \end{array}$$
 E.g. 4 [20]:
  (13) $$\begin{array}{c} min\;f_{4}\left(x_{1},x_{2}\right)=0.5+\frac{{sin}^{2}\sqrt{x_{1}^{2}+x_{2}^{2}}-0.5}{{\left[1+0.001\left(x_{1}^{2}+x_{2}^{2}\right)\right]}^{2}}\\ s.t.\;-100<x_{1},x_{2}<100. \end{array}$$

**Figure 1:**
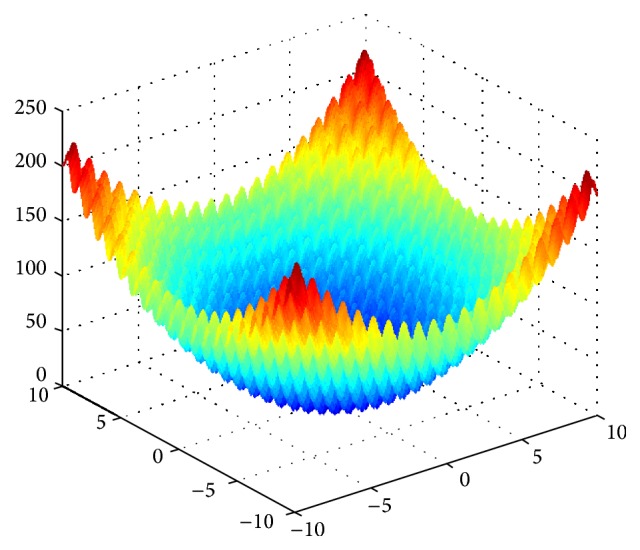
Image of function *f*_1_(*x*).

**Figure 2:**
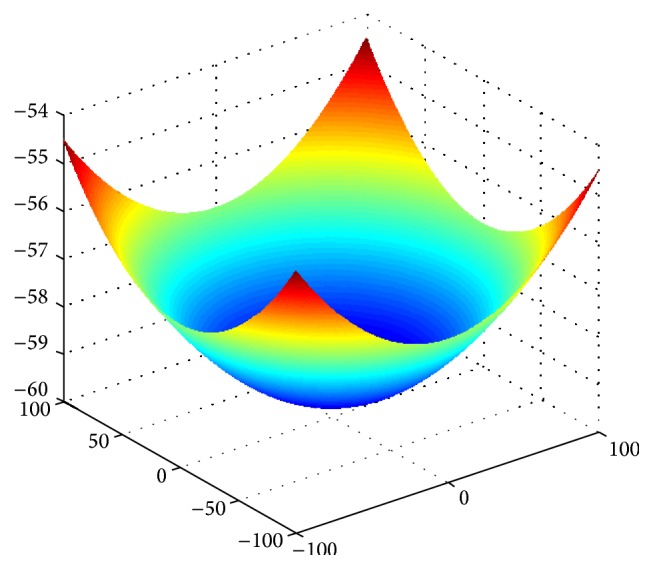
Image of function *f*_2_(*x*).

**Figure 3:**
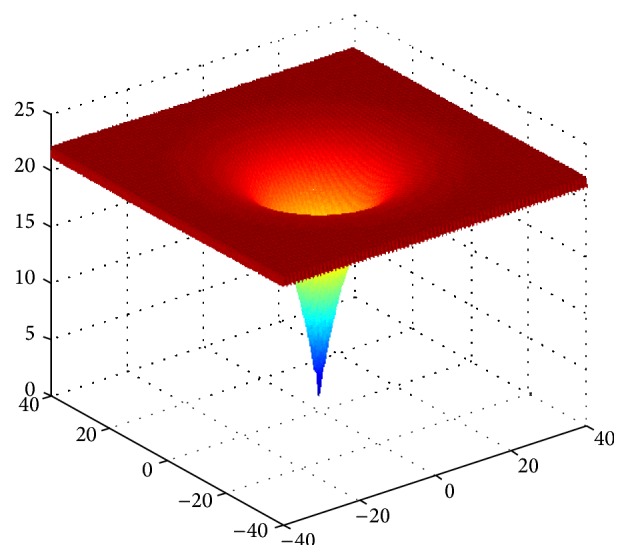
Image of function *f*_3_(*x*).

**Figure 4:**
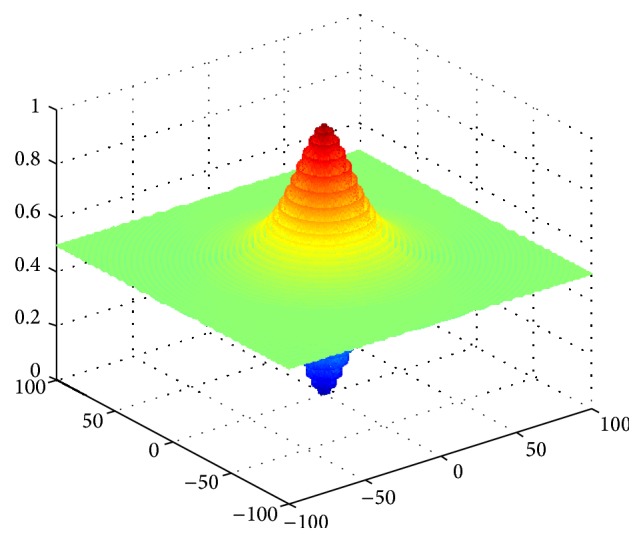
Image of function *f*_4_(*x*).

We can find the optimal value to be 0 through the analysis for function expression and the function image shown in Figures 1, 2, 3, and 4. The theoretical minimum value of these four multidimensional functions are 0 actually, where *f*_4_(*x*) is a two-dimensional function; other functions are all multidimensional functions. Use BAFSA, GAFSA, QAFSA, and IQAFSA to get minimum value of four different functions, respectively. Parameter settings are set as follows: fishnum = 40, try-number = 9, visual = 1, *δ* = 0.618, step_min⁡_ = 0.0002, *s* = 2, step = 0.5, and visual_min⁡_ = 0.001. The maximum iteration is set to be 1000 times and 1500 times. All algorithms run 50 times from different random initial population, respectively. The global average minimum value, the global minimum value, and standard deviation are to be as a measurement of the performance of algorithm, and the results are shown from Tables 2(a) and 2(b), where fun denotes function and dim⁡ denotes dimension; max⁡*T* is maximum evolution generation, *A*_min⁡_ is average minimum, *G*_min⁡_ is global minimum value, and *δ* is standard deviation, respectively.

**Figure 5:**
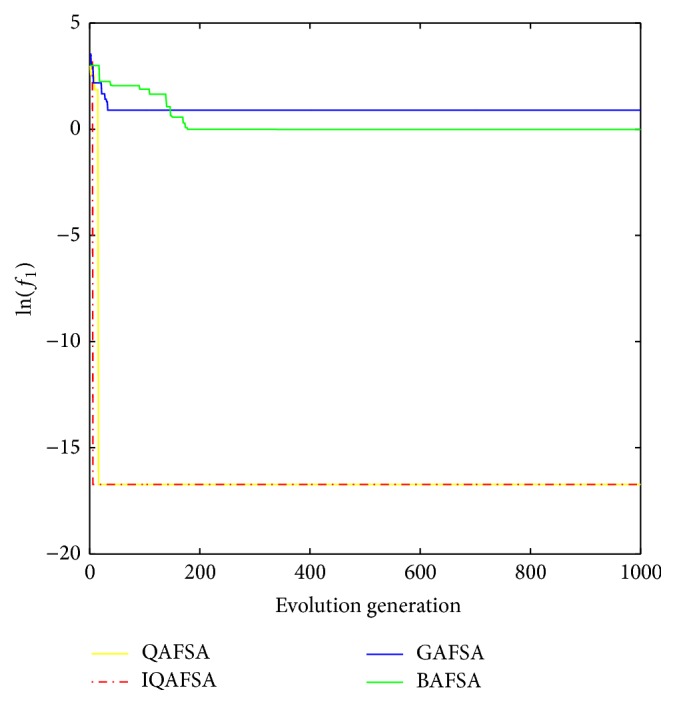
Average min evolution curve 3D function of *f*_1_(*x*).

**Figure 6 fig6:**
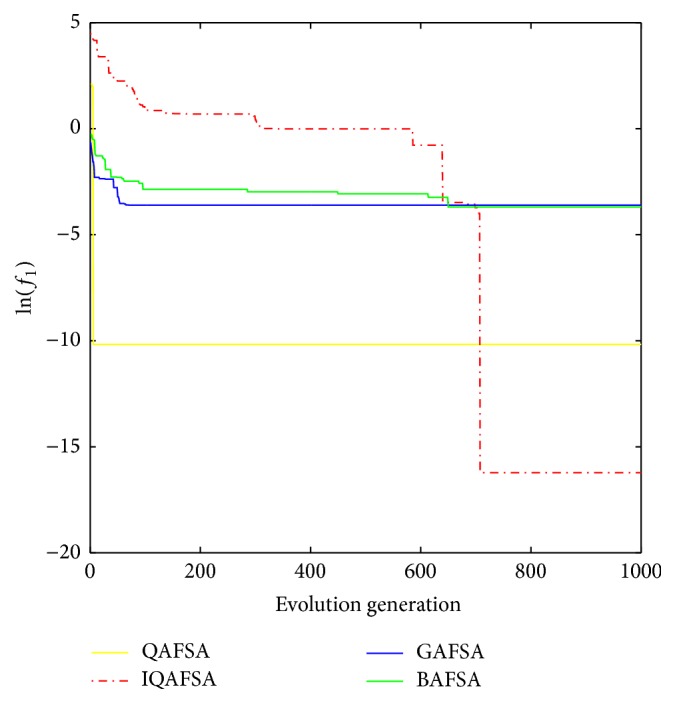
Average min evolution curve 5D function of *f*_1_(*x*).

**Figure 7 fig7:**
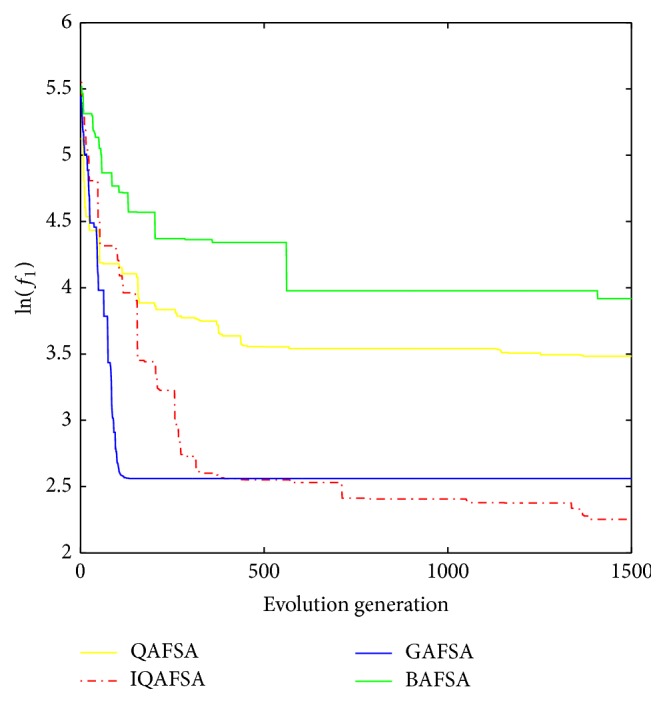
Average min evolution curve 10D function of *f*_1_(*x*).

**Figure 8 fig8:**
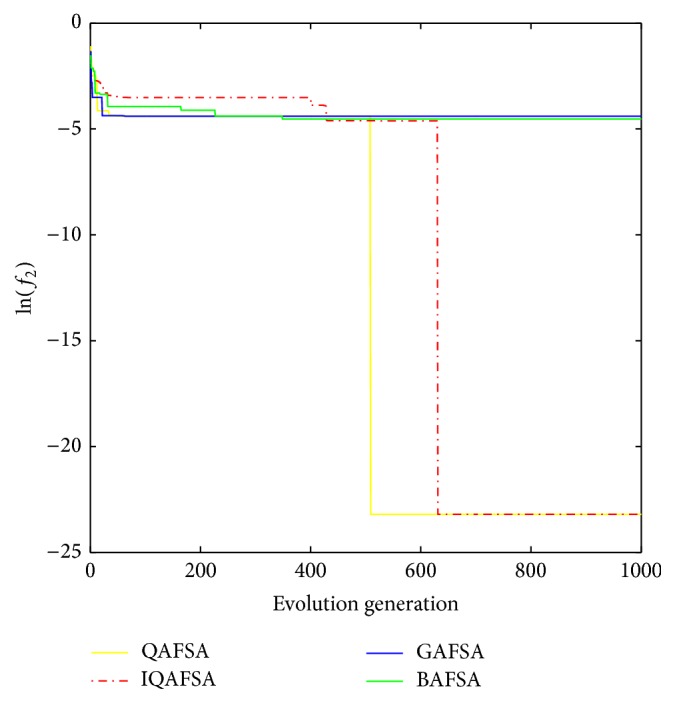
Average min evolution curve 3D function of *f*_2_(*x*).

**Figure 9 fig9:**
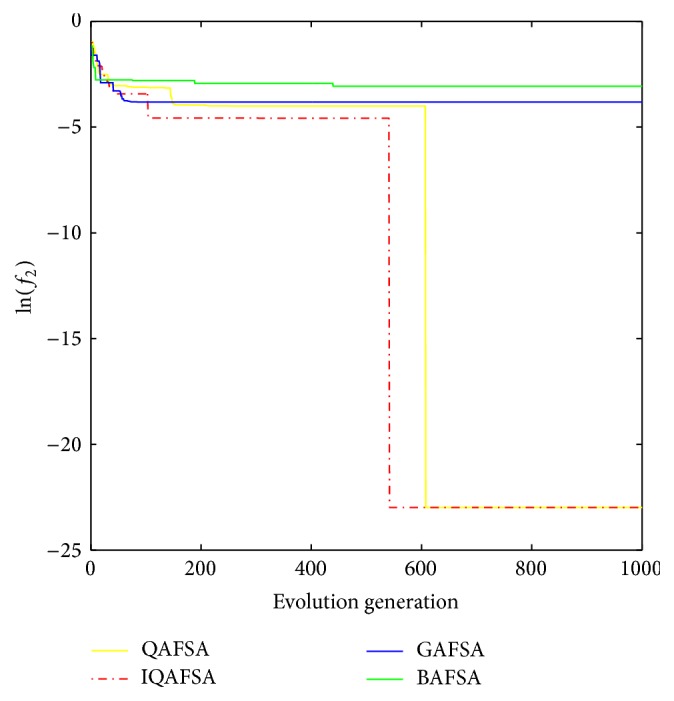
Average min evolution curve 5D function of *f*_2_(*x*).

**Figure 10 fig10:**
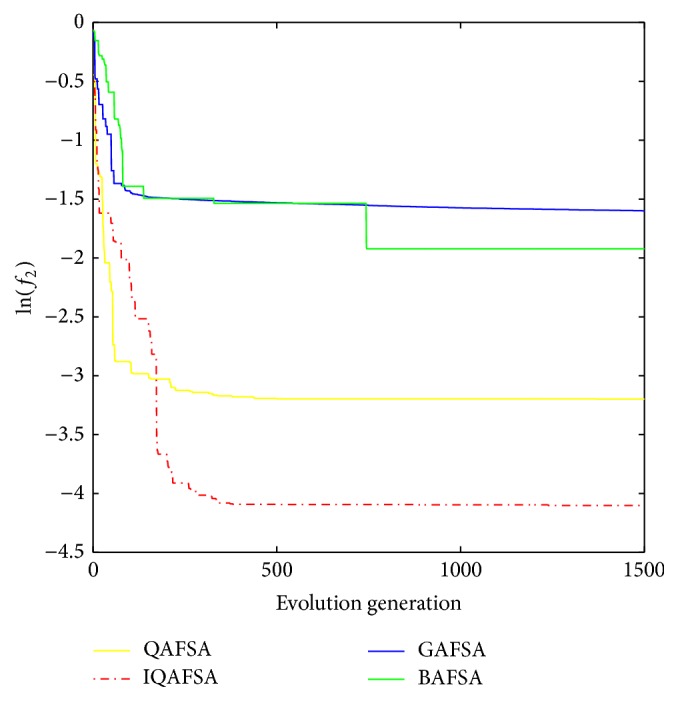
Average min evolution curve 10D function of *f*_2_(*x*).

**Figure 11 fig11:**
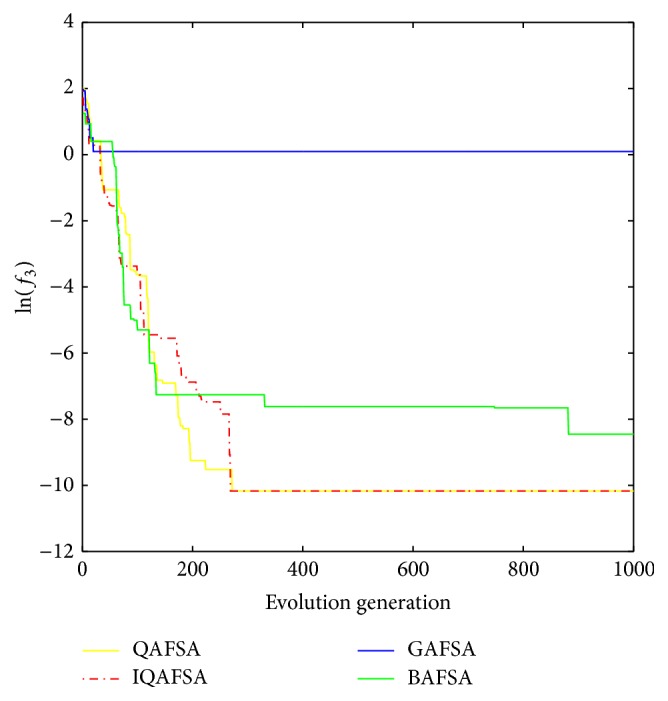
Average min evolution curve 3D function of *f*_3_(*x*).

**Figure 12 fig12:**
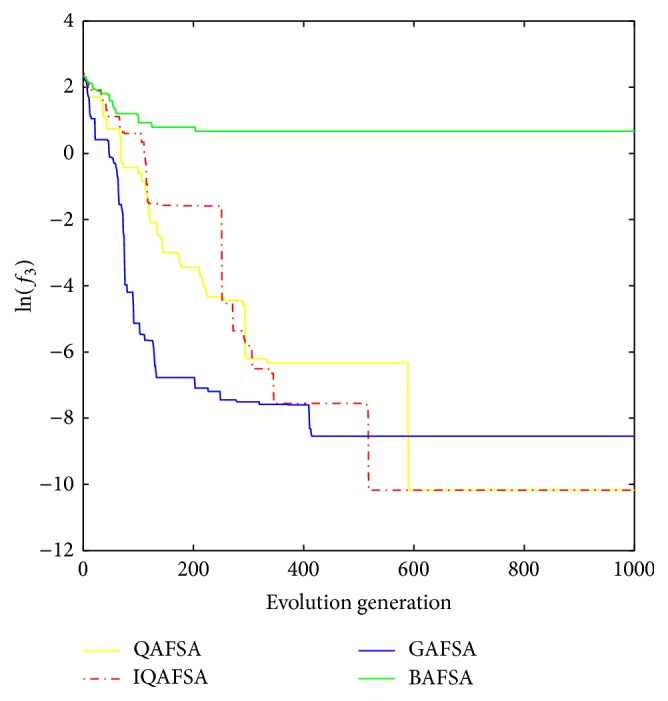
Average min evolution curve 5D function of *f*_3_(*x*).

**Figure 13 fig13:**
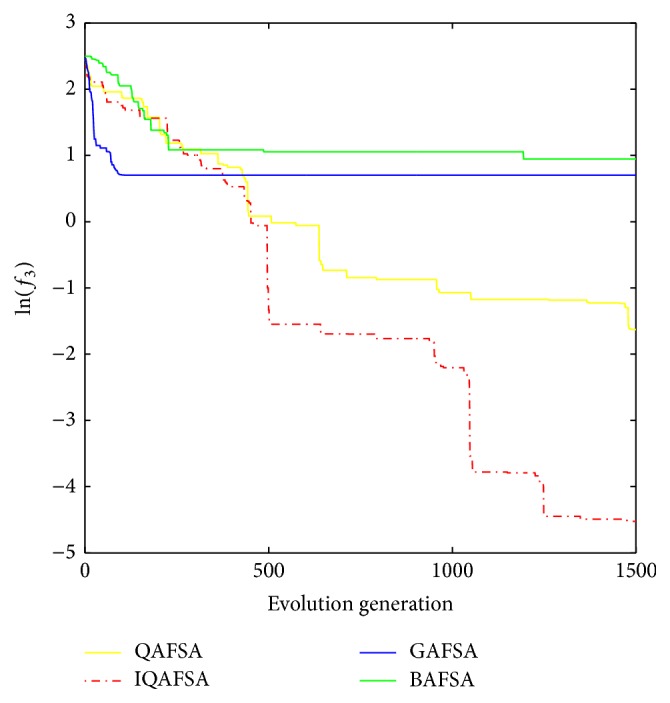
Average min evolution curve 10D function of *f*_3_(*x*).

**Figure 14:**
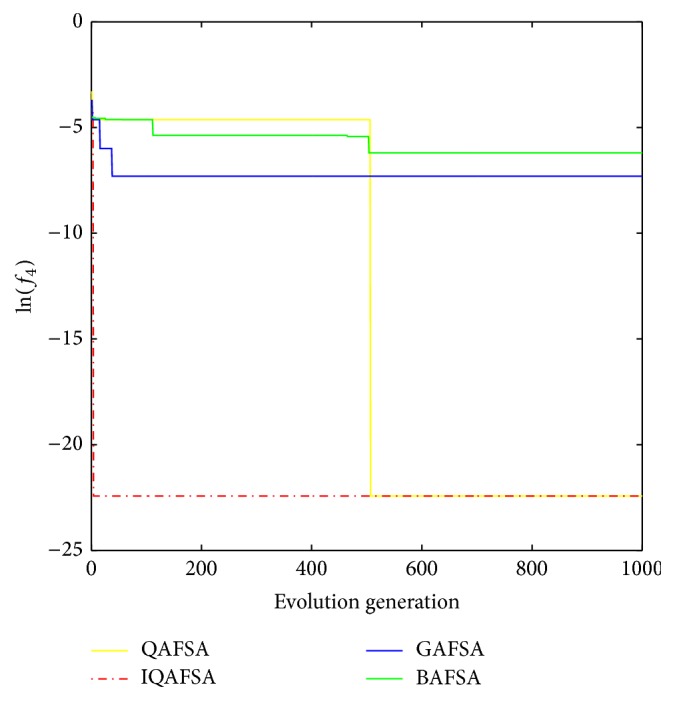
Average min evolution curve 3D function of *f*_4_(*x*).

This paper uses BAFSA, GAFSA, QAFSA, and IQAFSA for 50 times and obtains the average of the evolutionary curve and draws Figures 5 [Fig fig6] [Fig fig7] [Fig fig8] [Fig fig9] [Fig fig10] [Fig fig11] [Fig fig12] [Fig fig13]–14 of different dimension functions *f*_1_, *f*_2_, *f*_3_, and *f*_4_. In Table 2, the average minimum value, the global minimum value, and the global minimum of the four functions have been obtained under different dimensions and number of iterations by using BAFSA, GAFSA, QAFSA, and IQAFSA. In each figure, the ordinate is expressed by natural logarithm of optimal average of the function (i.e., ln(*f*)), and the abscissa is expressed by evolution generation; four different kinds of lines show the changing tendency of minimum value obtained by BAFSA, GAFSA, QAFSA, and IQAFSA with the increase of the number of iterations. From Table 2, it is clearly seen that the average optimization results and the optimal results of IQAFSA are much better than those of GAFSA, BAFSA, and QAFSA in different dimensions and number of iterations of each function. Meanwhile, the precision of the optimization results is greater than GAFSA, BAFSA, and QAFSA. Furthermore, most of standard deviations of IQAFSA are almost 0. In addition, from each figure, it is quite evident that stability of  IQAFSA is better than those of GAFSA, BAFSA, and QAFSA by comparing the standard deviation. With the increase of the dimension, the effect becomes more obvious compared with other algorithms. For example, the standard deviation of function *f*_1_ with 3D is 0 obtained by IQAFSA, but the others are usually bigger than 0. At the same time, in all figures, each optimal value is in the form of a logarithm in order to broaden the differences between different algorithms. From each figure, it is easily seen that convergence speed and optimization precision of IQAFSA are obviously better than those of GAFSA, BAFSA, and QAFSA. For example, Figure 8 shows that the optimal values obtained in the quickest way by IQAFSA are better than any other optimal values by other algorithms.

## 7. Distributed Network Considering DG

### 7.1. Formulation of Objective Function

The optimal configuration of the distribution networks with DG units is a nonlinear optimization problem. In this paper, the objective function of the nonlinear model is formulated with the aim of finding the minimum power loss. Thus, on the condition that the constraints below are considered, the objective function of the reconstruction is defined as

(14) $$\begin{array}{c} \underset{}{\min}f=\overset{n}{\underset{i=1}{\sum }}\frac{P_{i}^{2}+Q_{i}^{2}}{U_{i}^{2}}\cdot R_{i}, \end{array}$$

where *P*_*i*_  is real power load and *Q*_*i*_ is reactive power load of the node *i*, respectively. *U*_*i*_ is voltage of node *i*, and *R*_*i*_ is resistance of branch *i*, whilst *n* is the number of branches.

### 7.2. Constraints

(a) Voltage amplitude constraints are
  (15) $$\begin{array}{c} U_{min}\leq U_{i}\leq U_{max}. \end{array}$$
(b) Active power and reactive power constraints of the DG units are
  (16) $$\begin{array}{c} P_{DG_{i}}^{min}\leq P_{DG_{i}}\leq P_{DG_{i}}^{max},\\ Q_{DG_{i}}^{min}\leq Q_{DG_{i}}\leq Q_{DG_{i}}^{max}. \end{array}$$
(c) Current constraints are
  (17) $$\begin{array}{c} I_{i}^{min}\leq I_{i}\leq I_{i}^{max}. \end{array}$$
(d) Power flow equation constraints are
  (18) $$\begin{array}{c} P_{i}=V_{i}\overset{n}{\underset{j=1}{\sum }}V_{j}\left(G_{ij}\cos\delta_{ij}+B_{ij}\sin\delta_{ij}\right),\\ Q_{i}=V_{i}\overset{n}{\underset{j=1}{\sum }}V_{j}\left(G_{ij}\cos\delta_{ij}-B_{ij}\sin\delta_{ij}\right), \end{array}$$

where *U*_*i*_ is voltage of node *i*, *U*_min⁡_ is the minimum, and *U*_max⁡_ is the maximum of the node voltage, respectively. *P*_DG_*i*__ is active power and *Q*_DG_*i*__ is reactive power of DG_*i*_, respectively; *P*_DG_*i*__^min⁡^ is the minimum and *P*_DG_*i*__^max⁡^ is the maximum of the active power of DG_*i*_, whilst *Q*_DG_*i*__^min⁡^ is minimum of the reactive power of DG_*i*_ and *Q*_DG_*i*__^max⁡^ is the maximum. *I*_*i*_ is the current of the node *i*, *I*_min⁡_ is the minimum, and *I*_max⁡_ is the maximum of node current separately. *G*_*ij*_ is conductance and *B*_*ij*_ is susceptance between node *i* and node *j*. *δ*_*ij*_ is the voltage phase angle difference.

### 7.3. Handing of the DG in Calculating Load Flow

**Figure 15:**
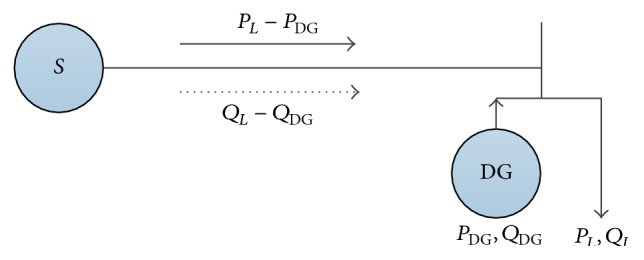
Basic configuration of distribution network after installing DG.

DG installed on the radial power distribution network can be simplified to a PV node, PQ node, or PI node. In this paper, DG is considered as a PQ node which has constant power factor. The characteristic of DG makes it an optional and alternative power supply normally so that it is usually installed on the location of the load. Assume that placements of all DG units are in the neighborhood of the load instead of line in the present paper. So, the basic configuration of the distribution network after installing DG is shown in Figure 15.

According to the comparison of active powers from the load and DG, there are three kinds of situations between load and distributed network when it comes to active power flow.

(1) If *P*_DG_*i*__ is greater than *P*_*L*_*i*__, the load of the node *i* can be considered as power supply outputting *P*_DG_*i*__ − *P*_*L*_*i*__ to distributed network, namely, to form reverse power flow.
(2) If *P*_DG_*i*__ is equal to *P*_*L*_*i*__, there is no active flow between distributed network and load.
(3) If *P*_DG_*i*__ is less than *P*_*L*_*i*__, the distributed network provides load with the active power of *P*_*L*_*i*__ − *P*_DG_*i*__.

**Figure 16:**
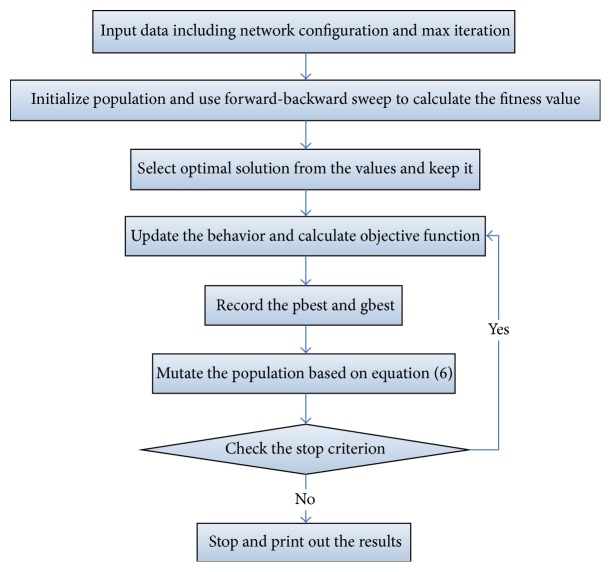
Flowchart of the proposed IQAFSA considering DG.

The route is shown in Figure 16.

### 7.4. Simulation

**Figure 17:**
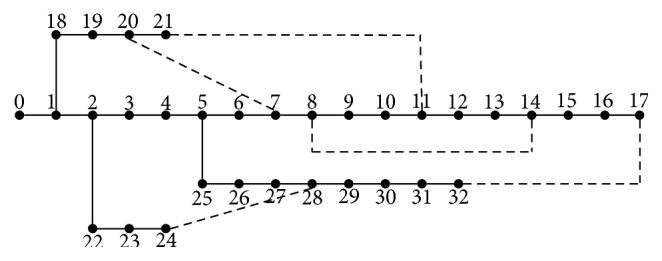
A 33-bus radial distribution network system.

**Figure 18:**
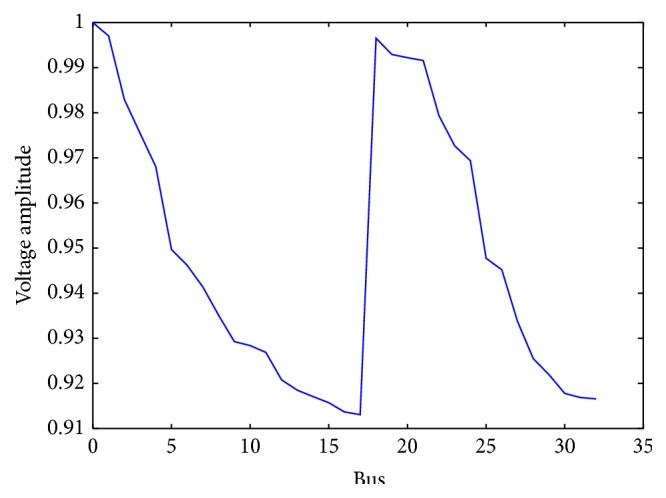
Voltage amplitude of IEEE33 nodes system.

IEEE33 nodes distributed network system [26] is adopted to take a simulation test in our work as shown in Figure 17. The rated voltage, total active power, and reactive power of system are 12.26 kV, 3715 kW, and 2300 kvar [27], respectively. Concurrently the active power loss is 201.715 kW and reactive power loss is 148.225 kW without DG units. The voltage amplitude of the system is shown in Figure 18. To verify feasibility and stability of IQAFSA and compare with BAFSA, GAFSA, and QAFSA, it is assumed that locations of DG units are fixed due to certain constraints such as power resources stability; buses 16, 17, and 32 whose voltage qualities are the lower are selected as DG location, and all of them are operated in a constant power factor mode, also known as the PQ node. It is equal to negative load which is installed in the neighborhood of the load.

Considering that decrease in total power loss depends on size and location of DG in this paper, four kinds of algorithms were used to determine the optimal size of DG on the condition that power loss of system is minimum. Finally, results were compared with each other. Some parameters are set as follows: fishnum = 40, try-number = 9, visual = 1, *δ* = 0.618, step = 0.5, visual_min⁡_ = 0.001, step_min⁡_ = 0.0002, *s* = 2. The max evolutional generations were set to 500 times and results were gotten in Tables 3–7.

Tables 3, 4, 5, and 6 provide simulation results carried out on the test network given in Figure 17, using BAFSA, GAFSA, QAFSA, and IQAFSA algorithms, respectively. Simulation results are obtained from MATLAB and include the best solutions, as provided in Table 7. It can be observed from Tables 3–6 that the sizes of DG units are maintained within permitted range of tolerance. Table 7 gives the system losses and processing time with four kinds of algorithms, and the minimum obtained by IQAFSA after comparing results with each other. Therefore, the minimum system loss and processing time are 45.0 kW and 0.02 s corresponding to the fact that outputs of buses 16, 17, and 32 are 441.4 kW, 250 kW, 1000 kW, respectively. It is shown that the optimal placement of DG units in system caused a larger reduction in power losses and less time with IQAFSA. In consideration of realistic factors, the sizes of buses 16, 17, and 32 were set to 500 kW, 300 kW, and 1000 kW.

**Figure 19:**
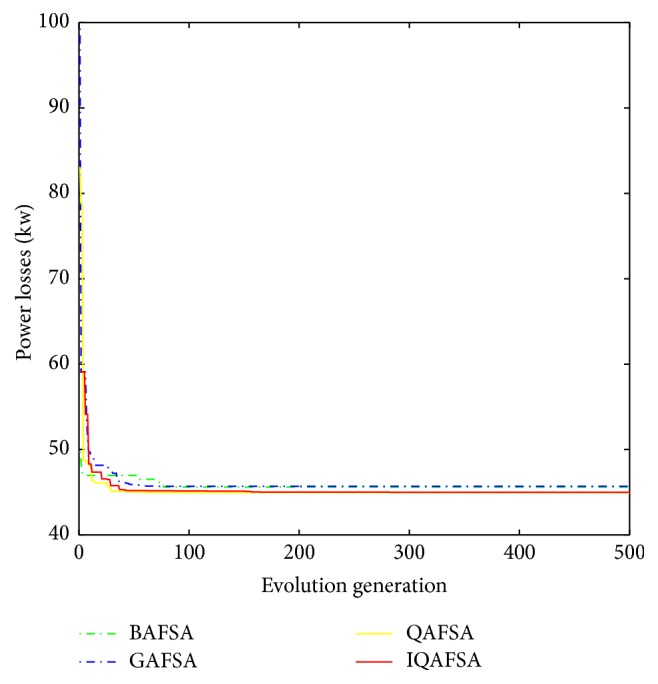
Convergence curves for four algorithms.

Convergence curves for four algorithms can also be used to show the capability and the speed of algorithm in obtaining the optimal results. By taking the best results that are given by four algorithms and plotting their best fitness value in the population for each iteration as shown in Figure 19, IQAFSA gives the faster results in reaching the global optimal solution and stayed there till the end of iterations. Furthermore, other algorithms failed to provide the global minimum and only reach the local minimum. Hence, it can be concluded that the proposed method in this paper results in a superior performance as compared to the existing methods such as BAFSA and QAFSA and also numerically converges more quickly than the traditional methods.

**Figure 20:**
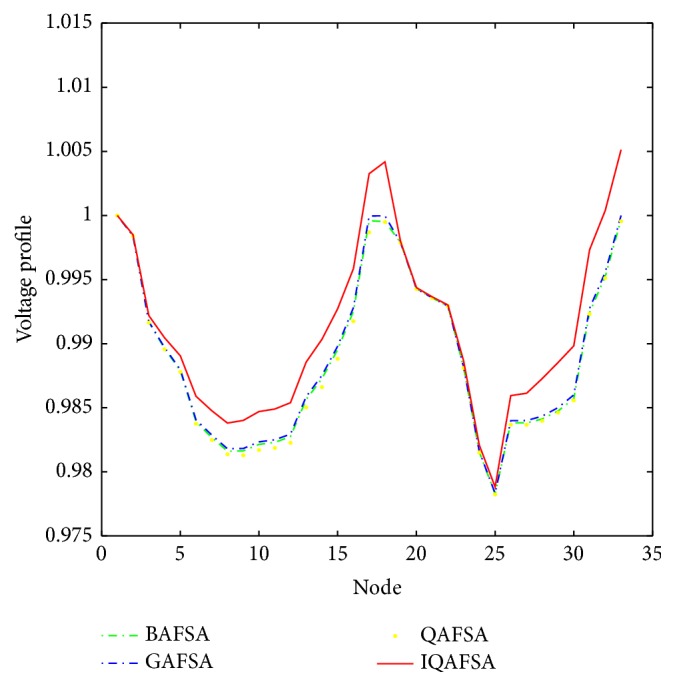
Voltage level comparison on IEEE33 nodes system.

Figure 20 shows the improvement in voltage profile under load models. As shown in Figure 20, the voltage at all buses after inserting DG units in system is higher than before, especially in the place of the bus where DG is located.

## 8. Conclusion 

In this paper, quantum computing is introduced on basis of artificial fish algorithm, and the improved quantum artificial fish algorithm is put forward. In the early stage of the artificial fish optimization, following behavior and preying behavior of artificial fish are added in the late stage, which improve convergence speed and optimization precision and enhance the diversity of population by implementation of the mutation after each iteration. Simulation results show that compared with GAFSA, BAFSA, and QAFSA, IQAFSA is more suitable for solving high dimensional and complex nonconvex programming. So the algorithm is more convergent. Finally, IQAFSA is applied to optimal configuration of DG in distributed network system and simulation results indicate that IQAFSA has great validity and feasibility. The theory research of IQAFSA is still in its infancy. In the future, using more test functions with higher dimension to verify performance of the proposed method is necessary. Convergence and stability of the algorithm remains to be proven so that more detailed work and further research from the theory are needed to expand.

## Figures and Tables

**Table 1:** Changing the angle value.

best_*i*_	*s*(*α*_*i*_, *β*_*i*_)			
	*α*_*i*_ *β*_*i*_ > 0	*α*_*i*_ *β*_*i*_ < 0	*α*_*i*_ = 0	*β*_*i*_ = 0
0	0	0	0	0
0	0	0	0	0
1	+1	−1	0	±1
1	−1	+1	±1	0
0	−1	+1	±1	0
0	+1	−1	0	±1
1	0	0	0	0
1	0	0	0	0

**(a):** 

fun	dim	max⁡*T*	BAFSA			GAFSA		
			*A*_min⁡_	*G*_min⁡_	*σ*	*A*_min⁡_	*G*_min⁡_	*σ*
*f*_1_	3	1000	1.49	1.303*e* − 4	0.62	1.49	6.526*e* − 6	2.01
	5	1000	9.391	5.434	2.20	2.9351	0.995	1.81
	10	1500	50.88	36.7075	7.82	20.844	2.985	12.1

*f*_2_	3	1000	0.007	8.301*e* − 4	4*e* − 3	8.5*e* − 3	2.99*e* − 10	7*e* − 3
	5	1000	0.0332	2.24*e* − 2	4*e* − 3	2.5*e* − 2	1.088*e* − 8	0.02
	10	1500	0.1584	0.1162	0.03	3.2433	2.3018	0.73

*f*_3_	3	1000	0.07	1.7*e* − 4	0.31	0.06	2.92*e* − 7	0.27
	5	1000	0.267	8.91*e* − 4	0.60	0.93	6.9*e* − 6	0.98
	10	1500	2.6388	2.1013	0.21	1.64	0.0011	0.93

*f*_4_	2	1000	0.0053	2.4*e* − 4	0.01	0.0039	6.3*e* − 15	0.05

**(b):** 

fun	dim	max⁡*T*	QAFSA			IQAFSA		
			*A*_min⁡_	*G*_min⁡_	*σ*	*A*_min⁡_	*G*_min⁡_	*σ*
*f*_1_	3	1000	5.013*e* − 6	5.413*e* − 8	0	5.413*e* − 8	5.413 − 8	0
	5	1000	0.3786	9.022*e* − 8	1.35	0.3786	9.022*e* − 8	1.35
	10	1500	10.154	2.701	8	9.4	1.804*e* − 7	5.7

*f*_2_	3	1000	8.34*e* − 11	8.3*e* − 11	0	8.34*e* − 11	8.3*e* − 11	0
	5	1000	6.074*e* − 3	1.0*e* − 10	0.016	6.074*e* − 3	1.0*e* − 10	0.016
	10	1500	3.211	2.121	0.21	0.1374	1.3*e* − 14	0.096

*f*_3_	3	1000	3.72*e* − 8	3.7*e* − 8	0	3.72*e* − 8	3.7*e* − 8	0
	5	1000	3.72*e* − 8	3.7*e* − 8	0	3.72*e* − 8	3.7*e* − 8	0
	10	1500	0.356	0.0128	0.53	0.0226	3.7*e* − 8	0.0278

*f*_4_	2	1000	0.9*e* − 10	1.6*e* − 16	0	1.6*e* − 16	1.6*e* − 16	0

**Table 3:** Suitable size for BAFSA.

Location of DG	16	17	32
Active power of DG/kW	416.6	211.9	965.1
Size of DG/kW	500	300	1000

**Table 4:** Suitable size for GAFSA.

Location of DG	16	17	32
Active power of DG/kW	574.1	93.1	938.5
Size of DG/kW	400	300	1000

**Table 5:** Suitable size for QAFSA.

Location of DG	16	17	32
Active power of DG/kW	187.5	500.0	1000.0
Size of DG/kW	300	500	1000

**Table 6:** Suitable size for IQAFSA.

Location of DG	16	17	32
Active power of DG/kW	441.1	250.0	1000
Size of DG/kW	500	300	1000

**Table 7:** Power losses and processing time.

	BAFSA	GAFSA	QAFSA	IQAFSA
Power loss/kW	46.0	45.4	45.9	45.0
Processing time/s	3.10	1.91	1.20	0.02
